# Effects of nebulized therapies on heat and moisture exchangers filters: a pilot study

**DOI:** 10.1186/s40635-025-00792-2

**Published:** 2025-08-08

**Authors:** Florian Blanchard, Cecilia Berardi, Lucie Collet, Jean-Michel Constantin

**Affiliations:** 1 GRC 29, AP-HP, DMU DREAM, Department of Anesthesiology and Critical Care, Pitié-Salpêtrière Hospital, Sorbonne University, Paris, France; 2 Istituto Di Anestesiologia E Rianimazione, Università Cattolica del Sacro Cuore, Largo F. Vito 1, 00168 Rome, Italy; 3 Department of Emergency, Intensive Care Medicine and AnestA. Gemelli IRCCS, Largo A. Gemelli 8, 00168 Rome, Italy; 4 Dipartimento di Scienze Biotecnologiche di Base, Cliniche Intensivologiche E Perioperatorie, Università Cattolica del Sacro Cuore, Largo F. Vito 1, 00168 Rome, Italy; 5 Present Address: Réanimation Chirurgicale Polyvalente, GH Pitié-Salpêtrière, 47-83 Boulevard de L’Hôpital, 75013 Paris, France

To the Editor,

During invasive mechanical ventilation (MV), heat and moisture exchangers (HMEs) are frequently used for humidification [1]. However, their presence poses a challenge during nebulization, as it may reduce drug delivery, increase filter saturation, and alter airway resistance (Raw) [2]. Clinicians often remove the HME during nebulization and replace it with an expiratory filter, exposing patients to circuit disconnection risks and increased costs [3]. Actual HME designs allow placement of the nebulizer downstream of the filter, with continuous gas flow, potentially avoiding HME removal. However, clinical data on the safety of this approach remain limited.

We conducted a prospective observational pilot study in a tertiary ICU to assess the impact of nebulized therapies on HME saturation and Raw in MV patients. Adult patients receiving vibrating mesh nebulization (Aerogen Solo®) of terbutaline (5 mg in a 2 mL solution) through an in-line configuration (ventilator → Y-piece → HME → nebulizer → patient) were included. Electrostatic HMEs (Covidien^®^) were weighed before and after each nebulization. In volume-controlled ventilation, Raw was measured under three conditions: with the patient’s HME in place (total Raw), without the HME (patient-specific Raw), and with a new HME (reference Raw). Filter-specific Raw was calculated as the difference between total and reference Raw. Ventilator settings were determined by the attending physician, except for bias flow, which was standardized at 3 L/min. This value corresponds to the continuous gas flow circulating through the circuit between breaths (bias or base flow) and is distinct from the inspiratory flow or minute ventilation. The primary outcome was HME weight variation as a surrogate for saturation; secondary outcomes included Raw changes and their progression over time.

Between July and October 2023, 19 patients received 83 nebulizations (Table 1). HME weight increased significantly after nebulization (median 25.5 g [IQR 25.2–26.2] vs 26.6 g [26.2–27.3], p < 0.001) and did not return to baseline between sessions, leading to progressive HME saturation (Fig. 1A and B). Airway resistance measurements (43 nebulizations in 15 patients) revealed no significant variation in total, patient-specific, or filter-specific Raw after nebulization (Fig. 1C, E, G). However, over time, total and patient-specific Raw showed a moderate increase, with no change in filter-specific Raw (Fig. 1D, F, H).

**Table 1:** Characteristic and clinical baseline patients’ data of the entire cohort

Variables	Entire cohort *n* = 19
Age, years, mean [SD]	59 [49–71]
Males, *n* (%)	15 (79%)
Female, *n* (%)	4 (21%)
BMI, kg/m^2^, mean [SD]	24.2 [22.1–27.3]
Arterial hypertension, *n* (%)	7 (37%)
Obstructive pulmonary disease, *n* (%)	4 (21%)
End stage heart failure, *n* (%)	2 (11%)
Smoking history, *n* (%)	12 (63%)
*Usual daily treatments*	
Inhaled beta-2-agonists, *n* (%)	2 (11%)
Oral corticosteroid, *n* (%)	3 (16%)
Home Oxygen therapy	1 (5%)
*Admission type*	
Medical, *n* (%)	8 (42%)
Elective surgery, *n* (%)	11 (58%)
SAPS II score at admission, median [IQR]	52 [45–61]
SOFA score at admission, median [IQR]	10 [5–12]
PaO2 over FiO2 at admission, median [IQR]	286 [237–318]
Mechanical ventilation at admission, *n* (%)	17 (89%)
Pulmonary infection at admission, *n* (%)	3 (16%)

*BMI* body mass index, *FiO2* fraction of inspired oxygen, *PaO2* partial pressure of arterial oxygen, *SAPS II* Simplified Acute Physiology Score II, *SOFA* Sequential Organ Failure Assessment

**Fig. 1:**
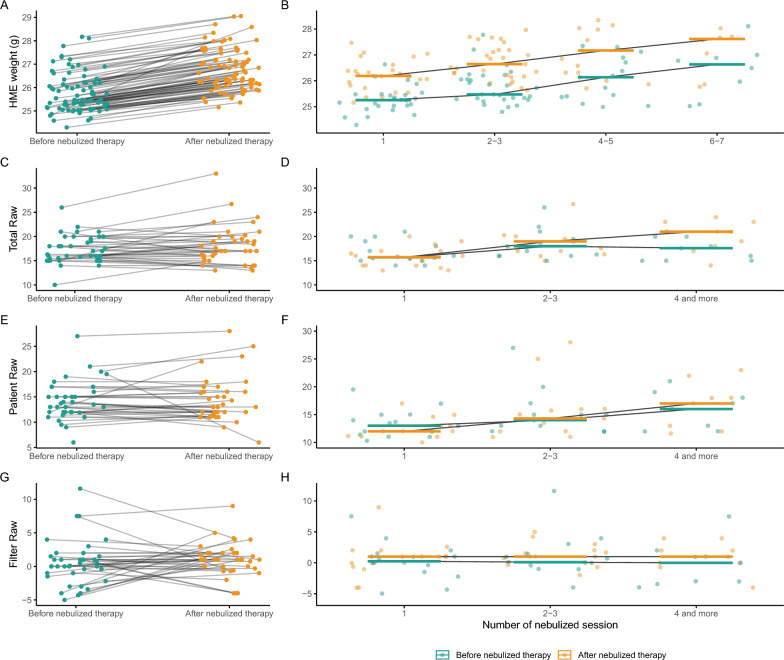
HME saturation and Raw variation: **A**. HME weight variation (i.e., HME saturation) before and after a nebulized therapy. HME weight increased after nebulized therapies (before: 25.5 g [25.2–26.2] vs after: 26.6 g [26.2–27.3], p < 0.001). **B.** HME saturation over the time (number of nebulized therapies). HME weight increased over the time (r = 0.46, p < 0.001 and r = 0.42, p < 0.001 before and after nebulized therapies, respectively). **C.** Total Raw (defined as the Raw with the patient HME) variation before and after a nebulized therapy. There was no variation of total Raw during a nebulized therapy (before: 16.6cmH2O/L/sec [15.4–19.4] vs after: 17.0 [15.6–19.9], p = 0.18). **D.** Total Raw variation over the time. Total Raw increased over the time (r = 0.17, p = 0.31 and r = 0.47, p = 0.0026 before and after nebulized therapies, respectively). **E.** Patient Raw (defined as the Raw without the patient HME) variation before and after a nebulized therapy. There was no variation of patient Raw during a nebulized therapy (before: 13.5cmH2O/L/sec [12.0–16.0] vs after: 13.0 [11.9–16.0], p = 0.94). **F.** Patient Raw variation over the time. Patient Raw increased over the time (r = 0.31, p = 0.062 and r = 0.54, p < 0.001 before and after nebulized therapies, respectively). **G.** Filter Raw (the difference of Raw with the patient HME and with a new HME) variation before and after a nebulized therapy. There was no variation of filter Raw during a nebulized therapy (before: 0.1cmH2O/L/sec [− 1.0–1.4] vs after: 1.0 [0.0–2.0], p = 0.40). **H.** Filter Raw variation over the time. Filter Raw did not increase over the time (r = − 0.16, p = 0.33 and r = 0.04, p = 0.81 before and after nebulized therapies, respectively). *HME* heat moisture exchangers filter, *Raw* airway resistances

Our findings suggest that nebulization through an HME is associated with measurable filter saturation but does not result in clinically significant airway resistance changes, even after repeated sessions. Interestingly, the increase in filter weight (~ 1 g per session) corresponds to approximately 50% of the nebulized solution, suggesting partial drug sequestration by the HME. This may explain the absence of a bronchodilator effect on patient-specific Raw. This suggests that maintaining the HME may be acceptable from a safety standpoint, but clinicians should be aware that doing so alters drug delivery dynamics. Therefore, rather than concluding that HME retention is feasible per se, our findings highlight that the HME significantly influences the quantity of drug reaching the lungs. If the HME is left in place, a higher nominal dose may be required to achieve therapeutic efficacy. Conversely, if the HME is removed, a greater amount of drug is delivered to the lungs. As nebulized antibiotic use grows—e.g., in VAP prevention protocols involving amikacin—the strategy of maintaining the HME in place may improve circuit integrity and cost-effectiveness, provided safety and drug delivery are ensured [4].

While limited by sample size, a single molecule, and one bias flow setting, this study is the first to evaluate real-world HME behavior during nebulization in MV patients. Further studies are warranted to evaluate efficacy and safety for other drug classes, especially inhaled antibiotics.

## Data Availability

Data collected for the study, including individual participant data, data dictionary defining each field in the set, and study protocol will be made available to others. Data will be communicated as de-identified participant data according to French law and will be available after publication of the manuscript. Data requests should be addressed to Dr. Florian Blanchard (florian.blanchard@aphp.fr) who will send the data after receipt of a signed data access agreement.
